# Supplementary figures and images for: Identification of cuproptosis and ferroptosis-related subtypes and development of a prognostic signature in colon cancer
Source: PLoS One. 2025 Jan 30;20(1):e0307013. doi: 10.1371/journal.pone.0307013 (PMC11781745; doi:10.1371/journal.pone.0307013)

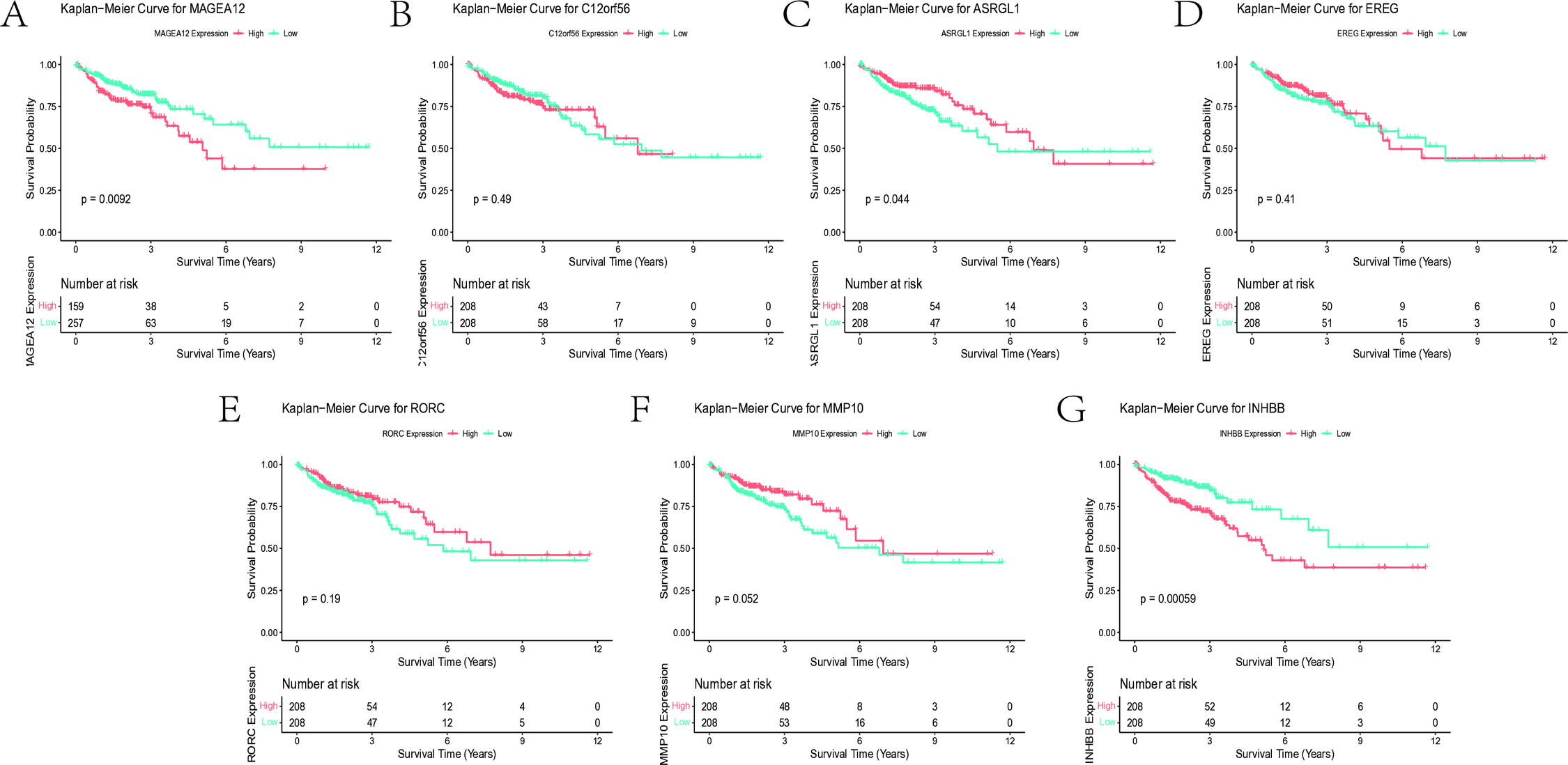

Supplement: S2 Fig — (TIF) [file pone.0307013.s002.tif]
