# Supplementary material for: Identification of cuproptosis and ferroptosis-related subtypes and development of a prognostic signature in colon cancer
Source: PLoS One. 2025 Jan 30;20(1):e0307013. doi: 10.1371/journal.pone.0307013 (PMC11781745; doi:10.1371/journal.pone.0307013)
Supplement: Attachment — Submitted filename: Response to reviewers.docx [file pone.0307013.s008.docx]

**Point-by-point response to reviewers**

We sincerely appreciate all the insightful comments and suggestions regarding our manuscript, which have greatly helped us to improve the quality of our manuscript. We have carefully considered each of the points raised and a point-by-point response to reviewers’ comments are listed below.

**Reviewer #1:**

*Comment 1:* *In the manuscript, the authors only used PCR technology to verify the results. Although the authors provided an effective prediction tool, I think the authors may need more experimental verification. Therefore, I think the manuscript needs to be revised before acceptance.*

**Response 1**: Thank you very much for your valuable suggestions. Relying solely on PCR validation results has several limitations; therefore, we further conducted immunohistochemical analyses on valuable genes identified in the immune characteristic and drug sensitivity analyses. The results are presented in the revised manuscript and figures.

**Reviewer #2:**

*Comment 1:* *It is a descriptive study without any mechanisms are validated or even proposed. The value of the results of this study is limited; it provided limited help for clinical diagnosis and treatment of colon cancer.*

**Response 1:** Thank you very much for your comments. Although our study did not validate the mechanisms of the genes, the immune characteristic and drug sensitivity analyses indicate that certain genes in the model have research value. Our future studies will further investigate these valuable genes, which may have significance for the development of targeted and chemotherapeutic agents. Additionally, our research established an effective predictive tool for estimating patient survival time, which can be utilized clinically to assess tumor malignancy.

*Comment 2: This study lacks innovation. Many studies have reported the role of ferroptosis and cuproptosis-related genes in cancer through bioinformatics analysis[1,2,3,4], and there have been study reporting the relationship between ferroptosis and cuproptosis-related genes and colon cancer[5]. So The present study lacks innovation.*

**Response 2:** We greatly appreciate your recognition of our research results. As you mentioned, the four articles [1, 2, 3, 4] respectively reported on the prognostic characteristics of cuproptosis- and ferroptosis-related genes in breast cancer, hepatocellular carcinoma, esophageal squamous cell carcinoma, and renal clear cell carcinoma. However, the focus of our study is on colon cancer. Although article [5] investigated the prognostic characteristics of cuproptosis- and ferroptosis-related genes in colorectal cancer, it differs from our study. Our research specifically focuses on colon cancer rather than colorectal cancer because we believe there are significant differences between colon cancer and rectal cancer in terms of anatomical location, clinical presentation, and case structure. For the first time, our study identifies subtypes in colon cancer by combining cuproptosis- and ferroptosis-related genes, and we developed a prognostic model based on the differential genes between the two subtypes. This approach allows us to more effectively identify potential genes related to both cuproptosis and ferroptosis. Additionally, we integrated immune cell and immune function gene sets from previous research on immune infiltration, leading to more comprehensive and precise results. Finally, while [5] used the HPA database for validation, our study employed qRT-PCR and IHC for validation. Therefore, we believe our study is more reliable compared to [5] and demonstrates a certain level of innovation.

*Comment 3: The research methods used in this study, such as Kaplan-Meier survival analysis, univariate, LASSO and multivariate Cox regression, GO and KEGG pathway enrichment analysis, nomogram, ESTIMATE, GSVA, are conventional and lack innovation.*

**Response 3:** Thank you very much for your comments. Although Kaplan-Meier survival analysis, univariate, LASSO, multivariate Cox regression and other methods are conventional, they are classic, well-established techniques that have been widely validated. While this approach may lack methodological innovation, it ensures the accuracy of the analytical results."

*Comment 4:For figure 11, why used qRT-PCR to detect genes expressions on colon cancer tissues and adjacent normal tissues, not IHC and western blot? In my opinion, IHC and western blot are better than qRT-PCR to detect genes expressions on colon cancer tissues and adjacent normal tissues.*

**Response 4**: Thank you very much for your valuable suggestions. Relying solely on qRT-PCR validation results has several limitations; therefore, we further conducted immunohistochemical analyses on valuable genes identified in the immune characteristic and drug sensitivity analyses. The results are presented in the revised manuscript and figures.

*Comment 5:For figure 11-O, there are 8 pairs of colon cancer tissues and adjacent normal tissues, not 10 pairs as described in the method.*

**Response 5**: Thank you very much for your question. Figure 11-O shows 10 pairs of colon cancer tissues and adjacent normal tissues. However, since the gene expression levels of some samples are similar, two points overlapped, making it appear as if there were only 8 pairs of tissue samples. The detailed situation can be seen in the original qRT-PCR data.

**References**

[1] Li J, Zhang W, Ma X, et al. Cuproptosis/ferroptosis-related gene signature is correlated with immune infiltration and predict the prognosis for patients with breast cancer. Front Pharmacol. 2023;14:1192434.
[2] Ma Q, Hui Y, Huang BR, et al. Ferroptosis and cuproptosis prognostic signature for prediction of prognosis, immunotherapy and drug sensitivity in hepatocellular carcinoma: development and validation based on TCGA and ICGC databases. Transl Cancer Res. 2023;12(1):46-64.
[3] Li J, Liu J, Li J, et al. A risk prognostic model for patients with esophageal squamous cell carcinoma basing on cuproptosis and ferroptosis. J Cancer Res Clin Oncol. 2023;149(13):11647-11659.
[4] Luo G, Wang L, Zheng Z, Gao B, Lei C. Cuproptosis-Related Ferroptosis genes for Predicting Prognosis in kidney renal clear cell carcinoma. Eur J Med Res. 2023;28(1):176.
[5] Li Y, Wang RY, Deng YJ, Wu SH, Sun X, Mu H. Molecular characteristics, clinical significance, and cancer immune interactions of cuproptosis and ferroptosis-associated genes in colorectal cancer. Front Oncol. 2022;12:975859.
