# Supplementary material for: Identification of cuproptosis and ferroptosis-related subtypes and development of a prognostic signature in colon cancer
Source: PLoS One. 2025 Jan 30;20(1):e0307013. doi: 10.1371/journal.pone.0307013 (PMC11781745; doi:10.1371/journal.pone.0307013)
Supplement: Attachment — Submitted filename: Response to reviewers .docx [file pone.0307013.s009.docx]

**Point-by-point response to reviewers**

We sincerely appreciate all the insightful comments and suggestions regarding our manuscript, which have greatly helped us to improve the quality of our manuscript. We have carefully considered each of the points raised and a point-by-point response to reviewers’ comments are listed below.

**Reviewer #2:**

*Comment 1: Figure 11 (A-G) and (H-N) are both labeled "TCGA Expression difference," and they do not include GEO database analysis as described in the results.*

**Response 1:** Thank you for pointing out the error in our study. This was due to our carelessness, which led to incorrect figure captions. We have corrected and re-uploaded the images for Figure 11 (A-G) and (H-N).

*Comment 2: The authors analyzed the expression differences of seven model-associated genes between tumor and normal tissues using the TCGA database, GEO database, and qRT-PCR. However, in the immunohistochemistry analysis, only 2 genes were examined. Please analyze all 7 genes using immunohistochemistry to ensure consistency with the other data.*

**Response 2:** We sincerely appreciate you pointing out the shortcomings in our study. Following your suggestions, we have completed the immunohistochemical analysis of the remaining five model-related genes, and the results are presented in Figure 12.

*Comment 3: The authors have already analyzed the expression differences of the seven model-associated genes between tumor and normal tissues. It is recommended to further investigate the correlation between these seven genes and patient prognosis to elucidate their impact on outcomes.*

**Response 3:** Thank you for your suggestions regarding this study. In fact, the study has already demonstrated the correlation between model-associated genes and prognosis through univariate Cox regression analysis. To further analyze the prognostic differences between groups with high and low expression of each model-associated genes, we plotted Kaplan-Meier (KM) curves for each model-associated gene, as shown in Supplementary Figure 2. We noticed that the results for certain genes differ from those obtained through univariate Cox regression analysis. We speculate that this discrepancy may be due to methodological differences. Kaplan-Meier analysis is a non-parametric method that considers only a single grouping variable’s impact on survival time without adjusting for other potential confounders. Additionally, the choice of grouping method may also influence the results of KM analysis.

*Comment 4: The introduction mentions that colorectal cancer exhibits different responses to treatment and prognoses due to the inherent heterogeneity of tumors, thus necessitating the search for new biomarkers to guide treatment. It then analyzes the cuproptosis and ferroptosis-related subtypes of colorectal cancer to clarify the tumor's intrinsic heterogeneity and guide treatment. However, there are currently no specific treatment methods targeting ferroptosis and cuproptosis in clinical practice for colorectal cancer. The author is requested to elaborate on why it is important to study cuproptosis and ferroptosis-related subtypes in colorectal cancer, the significance of this research, and its implications for future research or clinical practice.*

**Response 4**: We sincerely appreciate your comments on this study. Based on your suggestions, we have revised the Introduction section (paragraph 4) of the manuscript. Considering that cuproptosis- and ferroptosis-related subtypes have shown immune microenvironment characteristics and prognostic differences in many other tumors[1,2], we believe it is necessary to explore their features in colon cancer. Moreover, cuproptosis and ferroptosis are currently considered to have potential in overcoming tumor resistance[3]. Therefore, this study’s preliminary exploration of the interaction between cuproptosis and ferroptosis in the immune microenvironment of colon cancer also highlights their potential in colon cancer immunotherapy. Additionally, the drug sensitivity analysis performed in this study provides more therapeutic options for resistant tumors.

*Comment 5: The introduction points out the mutual dependence between ferroptosis and cuproptosis and then provides examples such as sorafenib and erastin, which can regulate the expression of the cuproptosis-related gene FDX1 and upregulate protein lipoylation. According to current studies, these mechanisms are related to cuproptosis. However, sorafenib and erastin primarily induce ferroptosis, and whether they can induce cuproptosis through these mechanisms remains unclear. Therefore, the mutual dependence between ferroptosis and cuproptosis cannot be definitively established. Please provide a detailed explanation of the interdependence between cuproptosis and ferroptosis, and elaborate on how these two modes of cell death interact or converge in colon cancer, thereby justifying the study’s rationale.*

**Response 5**: Thank you for your suggestions. Our wording may have been unclear, so we have made corresponding revisions to the Introduction section of the manuscript in response to your concerns. Based on previous studies, it is believed that there is some crosstalk between cuproptosis and ferroptosis[4]. Mitochondria, as a critical site for both ferroptosis and cuproptosis, have the TCA cycle as their convergence point. On one hand, the mitochondrial TCA cycle and electron transport chain play a central role in initiating ferroptosis by promoting mitochondrial membrane potential hyperpolarization and the accumulation of lipid peroxides[5]. On the other hand, the essential TCA cycle protein DLAT undergoes lipoylation mediated by FDX1, and the lipoylated protein’s binding with copper triggers copper toxicity[6]. Additionally, a recent study demonstrated that ferroptosis inducers sorafenib and erastin not only induce ferroptosis but also promote cuproptosis. The underlying mechanism involves inhibiting the degradation of FDX1 mediated by mitochondrial matrix-associated proteases and suppressing intracellular GSH synthesis[7]. This suggests that intracellular GSH synthesis may act as a common mediator between ferroptosis and cuproptosis[4].

*Comment 6: The discussion presents a broad overview of the findings, but fails to emphasize the uniqueness and specific contributions of this study to the existing body of knowledge on colon cancer. Please elaborate on the unique insights provided by this study in the field of colon cancer research and treatment, especially regarding the newly identified copper death and iron death related subtypes.*

**Response 6**: Thank you for your suggestions for improvement. We have enriched the corresponding content in the Discussion section of the manuscript. First, our study clarified that although there is no prognostic difference between cuproptosis- and ferroptosis-related subtypes in colon cancer, there are significant differences in immune cell infiltration and immune function. This indicates that different cuproptosis- and ferroptosis-related subtypes may exhibit varying responses to immunotherapy in colon cancer. Individualized immunotherapy strategies tailored to the immune cell infiltration characteristics of different subtypes could potentially enhance tumor treatment sensitivity to some extent. Second, our study identified several drugs that are sensitive to colorectal cancer and exhibit higher sensitivity in low-risk groups compared to high-risk groups. These drugs could be further investigated for their potential to be combined with existing chemotherapy regimens to overcome tumor resistance.

*Comment 7: The discussion mentions that certain genes or data exhibit inconsistencies when compared with the TCGA and GEO databases, but it does not delve into an analysis or provide hypotheses for these differences. Please provide a detailed explanation of these data discrepancies.*

**Response 7**: We sincerely appreciate your valuable comments. In the Discussion section of the manuscript, we have provided a detailed explanation regarding the differences in the analysis results between the TCGA and GEO databases, as well as the discrepancies between our qRT-PCR results and the public databases. Regarding the differences between the TCGA database and the GEO database, we believe the reasons are as follows: First, when the expression level of a gene is very low, the limitations of current technologies amplify measurement errors, ultimately leading to discrepancies between the two datasets. Second, the populations studied in the TCGA and GEO datasets differ. TCGA samples primarily represent populations from the Americas, whereas the GSE39582 cohort consists mainly of European populations, which may exhibit differences in gene expression. Third, the TCGA dataset uses RNA sequencing (RNAseq) data, while GSE39582 relies on microarray data. Compared to microarray, RNAseq is more accurate for detecting genes with low expression levels. Regarding the discrepancies between the qRT-PCR results and the public database, we believe this inconsistency may be attributed to the presence of a considerable amount of normal tissue even within macroscopically visible tumor samples. During qRT-PCR, the uncertainty in sample selection increases the likelihood of obtaining a significant proportion of normal tissue, leading to biased results. Subsequent immunohistochemistry confirmed that some genes are indeed highly expressed in cancer cells, while substantial amounts of normal tissue could still be observed in tumor tissue sections. Additionally, the limited sample size may have contributed to the differences in final statistical outcomes.

*Comment 8: The limitations section of the discussion is relatively brief and does not comprehensively address other potential shortcomings or areas for further research. Please offer a more in-depth exploration of the study's limitations and suggest directions for future research.*

**Response** 8: Thank you for your valuable comments on this study. Following your suggestions, we have conducted a more in-depth discussion of the study's limitations. First, although we constructed and validated a prognostic model based on cuproptosis and ferroptosis using public databases, additional prospective clinical data are needed to confirm the clinical significance of the model. Second, while we identified differences in immune cell infiltration components between the two cuproptosis- and ferroptosis-related subtypes, we did not explore the underlying mechanisms in detail. In particular, the regulatory roles of ASRGL1 and MMP10 in Th17 cells remain unclear and require further investigation through in vivo and in vitro experiments. Additionally, our study found that the expression of ASRGL1 and EREG can influence the sensitivity of colon cancer to certain antitumor drugs, and the specific mechanisms underlying these effects warrant further exploration. Finally, the antitumor drugs identified as potentially effective against low-risk colon cancer should be further validated in clinical practice to assess their efficacy.

**References**

[1] Yang BF, Ma Q, Hui Y, Gao XC, Ma DY, Li JX, et al. Identification of cuproptosis and ferroptosis-related subgroups and development of a signature for predicting prognosis and tumor microenvironment landscape in hepatocellular carcinoma. Transl Cancer Res. 2023;12(12):3327-45. Epub 20231227. doi: 10.21037/tcr-23-685. PubMed PMID: 38192999; PubMed Central PMCID: PMCPMC10774034.

[2] Li Y, Fang T, Shan W, Gao Q. Identification of a Novel Model for Predicting the Prognosis and Immune Response Based on Genes Related to Cuproptosis and Ferroptosis in Ovarian Cancer. Cancers (Basel). 2023;15(3). Epub 20230118. doi: 10.3390/cancers15030579. PubMed PMID: 36765541; PubMed Central PMCID: PMCPMC9913847.

[3] Wang JJ, Li JX, Liu J, Chan KY, Lee HS, Lin KN, et al. Interplay of Ferroptosis and Cuproptosis in Cancer: Dissecting Metal-Driven Mechanisms for Therapeutic Potentials. Cancers. 2024;16(3):19. doi: 10.3390/cancers16030512. PubMed PMID: WOS:001159184500001.

[4] Liu N, Chen MB. Crosstalk between ferroptosis and cuproptosis: From mechanism to potential clinical application. Biomed Pharmacother. 2024;171:16. doi: 10.1016/j.biopha.2023.116115. PubMed PMID: WOS:001154173100001.

[5] Gao M, Yi J, Zhu J, Minikes AM, Monian P, Thompson CB, et al. Role of Mitochondria in Ferroptosis. Mol Cell. 2019;73(2):354-63.e3. Epub 20181220. doi: 10.1016/j.molcel.2018.10.042. PubMed PMID: 30581146; PubMed Central PMCID: PMCPMC6338496.

[6] Tsvetkov P, Coy S, Petrova B, Dreishpoon M, Verma A, Abdusamad M, et al. Copper induces cell death by targeting lipoylated TCA cycle proteins. Science. 2022;375(6586):1254-+. doi: 10.1126/science.abf0529. PubMed PMID: WOS:000770386600033.

[7] Wang WK, Lu KZ, Jiang X, Wei Q, Zhu LY, Wang X, et al. Ferroptosis inducers enhanced cuproptosis induced by copper ionophores in primary liver cancer. J Exp Clin Cancer Res. 2023;42(1):12. doi: 10.1186/s13046-023-02720-2. PubMed PMID: WOS:001000971000001.
